# Xylazine induces dopamine release and augments the effects of fentanyl

**DOI:** 10.1172/JCI183354

**Published:** 2024-11-15

**Authors:** Joseph R. Trinko, Ethan Foscue, Edward M. Kong, Aakash Basu, Anouk M. Corstens, Summer L. Thompson, Alfred P. Kaye, Jane R. Taylor, Ralph J. DiLeone

**Affiliations:** 1 Department of Psychiatry, Yale University School of Medicine, New Haven, Connecticut, USA.; 2 Interdepartmental Neuroscience Program, Yale University, New Haven, Connecticut, USA.; 3 VA National Center for PTSD Clinical Neuroscience Division, West Haven, Connecticut, USA.; 4 Wu Tsai Institute, Yale University, New Haven, Connecticut, USA.

**Keywords:** Neuroscience, Addiction

## To the Editor:

Xylazine, an α2 adrenergic receptor agonist, was originally designated for veterinary multipurpose use as a sedative, analgesic, and muscle relaxant. Illicit use of xylazine started in Puerto Rico in the 2000s, with it becoming mixed as an adulterant with other drugs of abuse. Fentanyl-xylazine combinations began to proliferate in the northeastern US, with xylazine found in 90% of fentanyl samples, which was increasingly associated with higher percentages of fentanyl-associated deaths (1). The fentanyl-xylazine combination has complicated treatment of opioid use disorder (OUD), as high doses of xylazine can mimic some effects of opioids without being countered by naloxone/naltrexone administration (2, 3). Chronic injection of drugs laced with xylazine can also lead to tissue necrosis at the injection site, which may result in amputation and/or death (2). Preclinical work in animals investigating fentanyl-xylazine interactions is limited, and given xylazine’s impact on the OUD public health emergency, fundamental questions remain regarding how xylazine and fentanyl affect brain neurochemistry and OUD (4).

**Figure 1:**
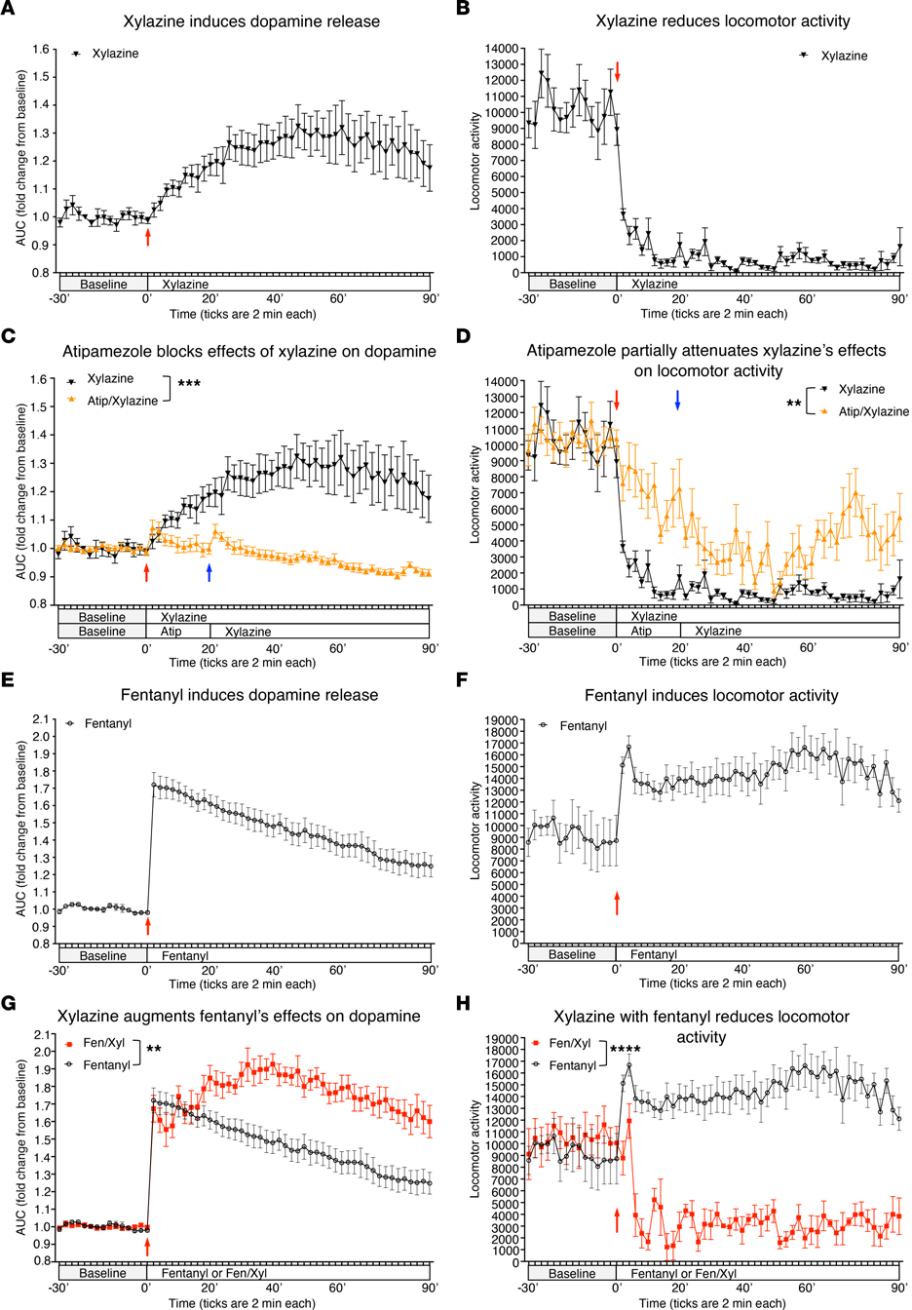
Xylazine induces dopamine release through an α2 adrenergic receptor effect and potentiates the neurochemical effects of fentanyl. (**A**) Xylazine (red arrow) significantly induced dopamine release (*n* = 6/group; repeated measures [RM] 1-way ANOVA main effect of time F_(44,220)_ = 4.499, ****P < 0.0001) and (**B**) reduced locomotor activity (*n* = 6/group; RM 1-way ANOVA main effect of time F_(44,220)_ = 3.452, ****P < 0.0001). (**C**) Pretreatment with the α2 adrenergic receptor antagonist atipamezole (red arrow) significantly blocked the effects of xylazine (blue arrow) on dopamine release (*n* = 6/group; 2-way RM-ANOVA main effect of treatment F_(1, 10)_ = 21.79, ***P = 0.0009; treatment × time interaction F_(34, 340)_ = 12.19, ****P < 0.0001 analyzing 70 minutes after atipamezole and xylazine treatments). (**D**) Atipamezole partially attenuated the effects on locomotor activity (*n* = 6/group; 2-way RM-ANOVA main effect of treatment F_(1,10)_ = 18.60, **P = 0.0015; treatment × time interaction F_(34,340)_ = 1.872, **P = 0.0030). (**E**) Fentanyl treatment (red arrow) induced significant dopamine release (*n* = 6/group; 1-way RM-ANOVA main effect of time F_(44, 220)_ = 34.52, ****P < 0.0001) and locomotor activity (*n* = 6/group; RM 1-way ANOVA main effect of time F_(44,220)_ = 1.828, **P = 0.0026) (**F**). (**G**) Coadministration of fentanyl and xylazine (red arrow) significantly augmented the effects of fentanyl alone on dopamine release (*n* = 6/group;2-way RM-ANOVA main effect of treatment F_(1,10)_ = 14.01, **P = 0.0038, and a significant interaction of treatment × time F_(44, 440)_ = 9.751, ****P < 0.0001), while locomotor activity was reduced compared to fentanyl alone (*n* = 4 combo, *n* = 6 fentanyl; 2-way RM-ANOVA main effect of treatment F_(1,8)_ = 60.65, ****P < 0.0001; treatment × time interaction F_(44,352)_ = 2.343, ****P < 0.0001) (**H**). All error bars are SEM.

We tested the effects of xylazine, with or without fentanyl, using fiber photometry to monitor dopamine release in the nucleus accumbens (NAc) of mice expressing the GRABDA2M sensor. This sensor has dopamine binding and modified EGFP domains, and it fluoresces relative to synaptic dopamine levels. Xylazine treatment (5 mg/kg, i.p.) significantly increased fluorescence, which peaked approximately 50 minutes after injection, suggesting increased dopamine release in the NAc (Figure 1A). In contrast, locomotor activity dropped to very low levels immediately after xylazine injection and remained significantly lowered throughout the session (Figure 1B). While reduced locomotor activity was expected considering the sedative properties of xylazine, the increase in extracellular dopamine was not anticipated.

To determine whether the effect of xylazine on dopamine was mediated directly by α2 adrenergic receptor signaling, we pretreated mice with a selective α2 adrenergic receptor antagonist. Atipamezole pretreatment (2 mg/kg, i.p.) 20 minutes prior to xylazine (5 mg/kg, i.p.) attenuated the effects of xylazine on dopamine release, confirming that this potential effect of xylazine on dopamine is dependent on α2 adrenergic signaling (Figure 1C). Locomotor activity was also significantly higher in these sessions compared with that of xylazine alone, consistent with α2 adrenergic–mediated sedative effects of xylazine (Figure 1D).

Opioids target dopamine circuits of the brain, suggesting a possible convergence of opioid and xylazine’s actions. As expected, fentanyl treatment (0.5 mg/kg, i.p.) resulted in a rapid increase in fluorescence (Figure 1E), with corresponding increases in locomotor activity (Figure 1F). Fentanyl and xylazine coadministration significantly increased fluorescence above that of the fentanyl-alone session (Figure 1G). This demonstrates an additive effect of fentanyl and xylazine on extracellular dopamine levels. In contrast, locomotor activity after combined fentanyl plus xylazine was significantly lower than with fentanyl alone (Figure 1H), consistent with xylazine’s sedative effects. Please refer to the Supplemental Methods for methodology description and supporting data as well as Supplemental Figure 1 for targeting and sample traces.

These present data demonstrate a clear and robust effect of xylazine on dopamine levels in the NAc of mice. This response is dependent upon the α2 adrenergic receptor and is additive when combined with fentanyl. While the target neurons and circuits mediating these changes are not yet clear, both direct midbrain and indirect circuit-mediated mechanisms are possible (5, 6). Effects of chronic xylazine, dose responses, and interactions with other factors such as age and sex will be important for future studies, as these data presented here showing effects of xylazine on dopamine signaling have previously unappreciated implications for how fentanyl-xylazine mixtures might impact the development and treatment of OUD.

Animal studies were approved by the IACUC.

## Supplementary Material

Supplemental data

Supporting data values

## Version 1 11/15/2024

Electronic publication
