# Supplementary material for: Xylazine induces dopamine release and augments the effects of fentanyl
Source: J Clin Invest. 2024 Nov 15;134(22):e183354. doi: 10.1172/JCI183354 (PMC11563672; doi:10.1172/JCI183354)
Supplement: Supplemental data [file jci-134-183354-s043.pdf]

Supplemental Methods and Figure Legend

Sex as a biological variable

Male and female mice were used for these experiments, however, no sex-specific conclusions are presented in these findings, as the sampling power needed for that level of investigation exceeds these preliminary experiments. Future experiments are needed to evaluate any sex-specific effects of xylazine using acute, as well as chronic treatment paradigms involving multiple doses which can better explore addiction-like models.

Animals

C57BL/6J young adult mice were purchased from the Jackson Laboratory (Bar Harbor, ME), and were at least 8 weeks of age at the time of surgery. Throughout the experiment, mice were group housed and maintained on a 12h light-dark cycle and fed *ad libitum* std. chow (#2018, Envigo LLC, Indianapolis, IN). pAAV-hsyn-GRAB-DA2m with a titer of $2.4x10^{13}$ was purchased from Addgene (Watertown, MA) (*1*). Mice were anesthetized with isoflurane and underwent intracranial viral infusion with GRAB-DA2m, delivering 0.5 µl unilaterally to the right nucleus accumbens (stereotaxic coordinates A/P +1.2, M/L +0.6, D/V -4.5 mm from bregma). A fiber optic canula (400 µm dia. fiber core, N.A. 4.8) was lowered to the same A/P and M/L coordinates, and D/V -4.2 mm from bregma), and then cemented in place. Mice were allowed to recover and for the virus to express for at least two weeks before any experimentation.

Fiber Photometry

The fiber photometry system consisted of a TDT RZ5 module (Tucker-Davis Technologies, Alachua FL), an LED driver with a 490 nm cube (all from Thorlabs, Newton NJ), and a photodetector (Newport #2151, Irvine CA). The emission spectrum passed through a 525 +/- 39

nm bandpass filter prior to photodetection, and these data were sampled by the TDT RZ5 module at a rate of 381.4697 Hz (*2*). After viral expression, mice were habituated to the fiber optic tether and to the testing room in order to reduce any novelty effects prior to experimentation. Test sessions consisted of initially photobleaching the fiber optic tether for at least 30 minutes at the beginning of each day of testing, prior to attaching a mouse. Mice were then tethered to a fiber optic cable (400 µm dia. core fiber, N.A. 4.8 Doric Lenses, Quebec Canada) and placed in a plastic mouse bucket with bedding for 30 minutes for general habituation with the LED turned off. Buckets and bedding were replaced when switching between cages of mice. After habituation, the LED was turned on and 30 minutes of baseline fluorescence was recorded before any injections. After treatment, the recording continued for 90 minutes. All mice went through all sessions for the treatments reported here, with at least four days of rest between sessions.

Drugs

Fentanyl Citrate (#F3886, Sigma-Aldrich) was diluted in saline. Xylazine (Rompun®) injectable 100 mg/ml, Dechra Veterinary Products) was diluted with saline. Atipamezole (#A9611, Sigma-Aldrich) was initially solubilized in DMSO and diluted with saline with a final concentration of 10% DMSO. In all experiments, drugs were administered intraperitoneally following the 30-min baseline period, except for the atipamezole pretreatment study, in which xylazine was administered 20 min later after atipamezole. The drug doses of 0.5 mg/kg (fentanyl citrate) and 5.0 mg/kg (xylazine) used here were similar to those used by other studies (*3–7*).

Locomotor Activity

To assess locomotor activity, we utilized DeepLabCut (*8*) a deep learning toolbox optimized for accurate pose estimation for a variety of lab animals from any view. A webcam was positioned above the mouse bucket and recorded a top-down view of the mouse at a rate of 30 frames per second. DeepLabCut used this input video and labeled each frame with a set of coordinates corresponding to "joints" or "critical points" of the animal according to a pretrained model. Each coordinate has a corresponding confidence level from 0-1 on how confident the algorithm's pose estimation is for that point.

MATLAB analysis

Data for the fiber photometry setup was collected as intensity from recorded filtered emission spectrum generated by the 490 nm wavelength. Recordings were continuous throughout the session, and fluorescent data recorded while investigators were in the room or handling the mice were removed from the data set prior to any normalization and baseline calculation. Handling typically did not exceed one minute. After splicing out the signal during handling/treatment, the average signal during the pre-drug baseline was calculated and used to calculate fold-change in fluorescence for the entire session. Area under the curve was then calculated for this fold change in two-minute bins. This method of using just a single signal for analysis without a valid isosbestic control is similar to that used by others for GRAB sensors (*1*, *9–11*). Shown in the main figure are AUCs for fold change in two-minute bins. Shown in the supplemental figure are normalized sample fluorescent traces (fold change) from the different conditions (all from the same mouse).

Using the data collected from DeepLabCut, we calculated the overall locomotor activity of the mouse over time. MATLAB was used to calculate the distance between two points in

between two consecutive frames for every frame and summed together into two-minute bins for each recorded period. Any points with a confidence level lower than 95 (as determined by DeepLabCut) were filtered out of the calculations as “0” distance covered. A total of three “critical points” from these data were used to interpret the movement of the mouse: the center point, right shoulder, and left shoulder. The summed movement of these points best reflected the overall locomotion of the mouse.

Immunofluorescence histology

Mice underwent deep anesthesia and then perfusion with 10% formalin prior to extracting the brains. The brains were postfixed in 10% formalin for two days, followed by cryoprotection in 30% sucrose in 1X PBS. After cryoprotection, brains were sectioned and 35 µm sections were stored in 1X PBS with 0.01% sodium azide. NAc sections were washed in 1X PBS, blocked for 90 minutes (1X PBS with 3% donkey serum, 0.3% Triton X-100), and then incubated overnight in blocking buffer containing chicken anti-GFP diluted 1:500 (Abcam Inc. #ab13970). After washing with 1X PBS, sections were incubated with fluorescent secondary donkey anti-chicken 488-conjugated antibody (Jackson ImmunoResearch Inc. #703-545-155) diluted 1:200 in 1X PBS for 2 hours and washed again in 1X PBS. Sections were slide mounted for imaging. The Franklin and Paxinos mouse brain atlas was used to identify viral spread and canula placement which are depicted in the supplemental figure, modified with permission from Elsevier, license ID 1476322-1 (*12*).

Statistics

GraphPad Prism 10 was used for statistical analysis using the fold change values. One-way repeated measures (RM)-ANOVAs were used for assessing changes over time for

experiments with just one group, while two-way RM-ANOVAs were used for comparing across treatments. An alpha threshold of 0.05 was used for statistical significance. Baselines were not included in the statistical analysis.

Study approval

All animal care and experimental procedures were conducted in accordance with the Yale University School of Medicine Institutional Animal Care and Use Committee (IACUC) guidelines.

Data availability

All MATLAB code will be made accessible upon request through the corresponding author, and supporting data for the main figure can be found in the Supporting Data Values file.

Acknowledgements

This work was funded in part by the State of Connecticut, Department of Mental Health and Addiction Services, but this publication does not express the views of the Department of Mental Health and Addiction Services or the state of Connecticut. S.L.T was supported by K99AA029454. The views and opinions expressed are those of the authors. A.P.K. has received research funding from Freedom Biosciences and Transcend Therapeutics, and has filed a provisional patent application related to novel pharmacological treatments in psychiatric disorders.

Supplemental Figure 1: 1A) Panels illustrating medial/lateral and dorsal/ventral targeting assessment for the approximate spread of the virus, as well as approximate canula placements for all six mice used in this study. Mouse brain stereotaxic graphics are used with permission from Elsevier. **1B)** Sample immunofluorescence showing eGFP expression in the NAc (at 10X

magnification) with key regions outlined. **1C-F)** Normalized sample fluorescent traces from a single mouse from xylazine alone, atipamezole prior to xylazine, fentanyl alone, and combination of fentanyl xylazine sessions. Red arrows indicate acute treatments corresponding to the panel titles, and the blue arrow depicts xylazine after atipamezole treatment.

References

1. F. Sun, J. Zhou, B. Dai, T. Qian, J. Zeng, X. Li, Y. Zhuo, Y. Zhang, Y. Wang, C. Qian, K. Tan, J. Feng, H. Dong, D. Lin, G. Cui, Y. Li, Next-generation GRAB sensors for monitoring dopaminergic activity in vivo. *Nat Methods* **17**, 1156–1166 (2020).

2. C. W. Bond, R. Trinko, E. Foscue, K. Furman, S. M. Groman, J. R. Taylor, R. J. DiLeone, Medial Nucleus Accumbens Projections to the Ventral Tegmental Area Control Food Consumption. *J Neurosci* **40**, 4727–4738 (2020).

3. S. N. Khatri, S. Sadek, P. T. Kendrick, E. O. Bondy, M. Hong, S. Pauss, D. Luo, T. E. Prisinzano, K. E. Dunn, J. A. Marusich, J. S. Beckmann, T. D. Hinds, C. D. Gipson, Xylazine suppresses fentanyl consumption during self-administration and induces a unique sex-specific withdrawal syndrome that is not altered by naloxone in rats. *Exp Clin Psychopharmacol*, doi: 10.1037/pha0000670 (2023).

4. C. M. St. Onge, J. R. Canfield, A. Ortiz, J. E. Sprague, M. L. Banks, Xylazine does not enhance fentanyl reinforcement in rats: A behavioral economic analysis. *Drug and Alcohol Dependence* **258**, 111282 (2024).

5. P. Haouzi, M. McCann, N. Tubbs, Control of Homeostasis: Respiratory effects of low and high doses of fentanyl in control and β-arrestin 2-deficient mice. *Journal of Neurophysiology* **125**, 1396 (2021).

6. S. J. Harp, M. Martini, W. Rosenow, L. D. Mesner, H. Johnson, C. R. Farber, E. F. Rissman, Fentanyl-induced acute and conditioned behaviors in two inbred mouse lines: potential role for Glyoxalase. *Physiol Behav* **243**, 113630 (2022).

7. J. P. Anand, B. T. Boyer, H. I. Mosberg, E. M. Jutkiewicz, The Behavioral Effects of a Mixed Efficacy Antinociceptive Peptide, VRP26, Following Chronic Administration in Mice. *Psychopharmacology (Berl)* **233**, 2479–2487 (2016).

8. A. Mathis, P. Mamidanna, K. M. Cury, T. Abe, V. N. Murthy, M. W. Mathis, M. Bethge, DeepLabCut: markerless pose estimation of user-defined body parts with deep learning. *Nat Neurosci* **21**, 1281–1289 (2018).

9. A. Basu, J.-H. Yang, A. Yu, S. Glaeser-Khan, J. A. Rondeau, J. Feng, J. H. Krystal, Y. Li, A. P. Kaye, Frontal Norepinephrine Represents a Threat Prediction Error Under Uncertainty. *Biological Psychiatry* **96**, 256–267 (2024).

10. J. Feng, H. Dong, J. E. Lischinsky, J. Zhou, F. Deng, C. Zhuang, X. Miao, H. Wang, G. Li, R. Cai, H. Xie, G. Cui, D. Lin, Y. Li, Monitoring norepinephrine release *in vivo* using next-generation GRABNE sensors. *Neuron* **112**, 1930-1942.e6 (2024).

11. L. Li, A. N. Rana, E. M. Li, J. Feng, Y. Li, M. R. Bruchas, Activity-dependent constraints on catecholamine signaling. *Cell Rep* **42**, 113566 (2023).

12. K. B. J. Franklin, G. Paxinos, *The Mouse Brain in Stereotaxic Coordinates* (Academic Press, San Diego, ed. 3, 2008).

Supplemental Figure 1.

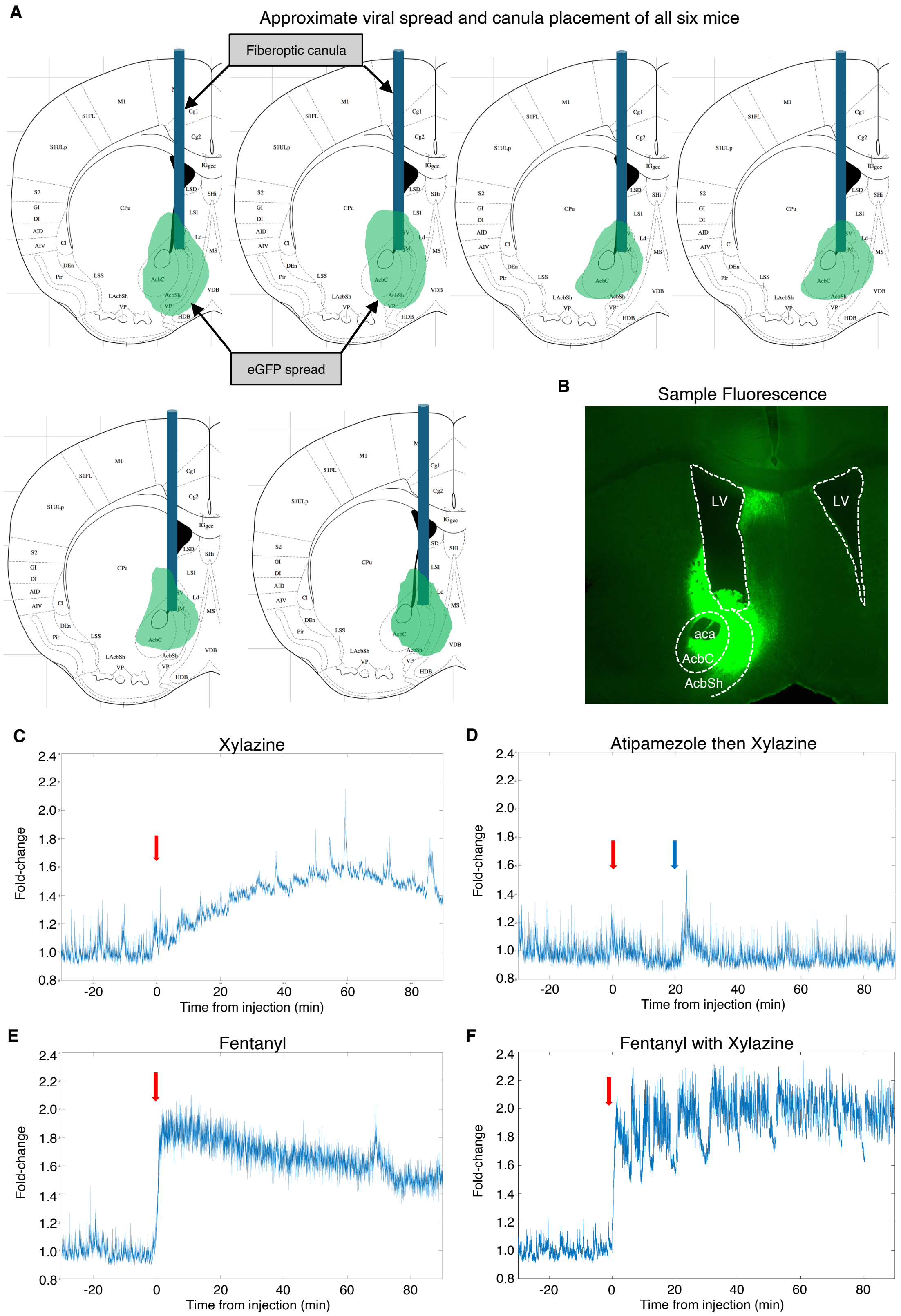

Supplemental Figure 1: 1A) Panels illustrating medial/lateral and dorsal/ventral targeting assessment for the approximate spread of the virus, as well as approximate canula placements for all six mice used in this study. Mouse brain stereotaxic plates are used with permission from Elsevier. **1B)** Sample immunofluorescence showing eGFP expression in the NAc (at 10X magnification) with key regions outlined. **1C-F)** Normalized sample fluorescent traces from a single mouse from xylazine alone, atipamezole prior to xylazine, fentanyl alone, and combination of fentanyl xylazine sessions. Red arrows indicate acute treatments corresponding to the panel titles, and the blue arrow depicts xylazine after atipamezole treatment.
